# Accumulating Evidence and Research Organization (AERO) model: a new tool for representing, analyzing, and planning a translational research program

**DOI:** 10.1186/1745-6215-14-159

**Published:** 2013-05-30

**Authors:** Spencer Phillips Hey, Charles M Heilig, Charles Weijer

**Affiliations:** 1 Studies for Translation, Ethics, and Medicine (STREAM) Group, Biomedical Ethics Unit, McGill University, Montreal, QC H3A 1X1, Canada; 2, Centers for Disease Control and Prevention, Division of Tuberculosis Elimination, 1600 Clifton Rd, NE, MS E10, Atlanta, GA, 30333, USA; 3 Rotman Institute of Philosophy, Department of Philosophy, Western University, 1151 Richmond Street, London, ON, N6A 5B8, Canada

**Keywords:** Translational medicine, Research efficiency, Graph-theoretic model, Robustness, Moxifloxacin, Tuberculosis, Research coordination, Research planning, Decision-making

## Abstract

**Background:**

Maximizing efficiency in drug development is important for drug developers, policymakers, and human subjects. Limited funds and the ethical imperative of risk minimization demand that researchers maximize the knowledge gained per patient-subject enrolled. Yet, despite a common perception that the current system of drug development is beset by inefficiencies, there remain few approaches for systematically representing, analyzing, and communicating the efficiency and coordination of the research enterprise. In this paper, we present the first steps toward developing such an approach: a graph-theoretic tool for representing the Accumulating Evidence and Research Organization (AERO) across a translational trajectory.

**Methods:**

This initial version of the AERO model focuses on elucidating two dimensions of robustness: (1) the consistency of results among studies with an identical or similar outcome metric; and (2) the concordance of results among studies with qualitatively different outcome metrics. The visual structure of the model is a directed acyclic graph, designed to capture these two dimensions of robustness and their relationship to three basic questions that underlie the planning of a translational research program: What is the accumulating state of total evidence? What has been the translational trajectory? What studies should be done next?

**Results:**

We demonstrate the utility of the AERO model with an application to a case study involving the antibacterial agent, moxifloxacin, for the treatment of drug-susceptible tuberculosis. We then consider some possible elaborations for the AERO model and propose a number of ways in which the tool could be used to enhance the planning, reporting, and analysis of clinical trials.

**Conclusion:**

The AERO model provides an immediate visual representation of the number of studies done at any stage of research, depicting both the robustness of evidence and the relationship of each study to the larger translational trajectory. In so doing, it makes some of the invisible or inchoate properties of the research system explicit – helping to elucidate judgments about the accumulating state of evidence and supporting decision-making for future research.

## Background

Maximizing efficiency across a drug development trajectory is important for drug developers, policymakers, and human subjects. Limited resources and the ethical imperative of risk minimization demand both that researchers design and plan their studies to maximize the knowledge gained per patient-subject enrolled and that funders and ethical review boards hold them to this standard.

Unfortunately, this standard is often not met. The infamous failure of torcetrapib at phase 3 illuminated inefficiencies in cardiovascular drug development [1-3]. In cancer, the cases of sunitinib for treating hepatic cancer and bevacizumab for treating gastric cancer were both examples of drugs that advanced into phase 3 testing without supporting phase 2 evidence [4]. Across the entire spectrum of drug development, nearly one-third of the drugs abandoned at phase 2 are considered failures not for lack of efficacy, but for ‘strategic’ reasons [4]. Even within a single company, as Pfizer’s internal review showed, almost half of their phase 2 proof-of-concept studies (43%) failed to test the target mechanism of action adequately [5].

These problems all contribute to the common perception that the current system of drug development is beset by inefficiencies [4,6,7], and the growing number of calls for greater coordination across the drug development enterprise [8-10]. Yet, there remain no systematic approaches for representing, analyzing, and communicating the efficiency and coordination of the research enterprise. The available techniques of systematic review and statistical meta-analysis can only tell a part of this story – elucidating partial cross sections of the evidence – but they do not offer any synthesis for the accumulating state of evidence across the entire translational trajectory. For example, a meta-analysis is well suited to identifying trends across a series of similar trials, but it is useless for understanding transitions between the different phases of research. How did the research program progress from pre-clinical to clinical studies? How did the results of phase 1 studies compare with phase 2? What does the failure of translation from pre-clinical to clinical tell us about what we ought to do next? These kinds of questions are entirely consistent with the aim of comprehensively understanding the translational research enterprise, but they are not questions for which a statistical meta-analysis is helpful.

In this paper, we present a first step toward developing a broader, more comprehensive approach to representing and understanding a translational research program: A graph-theoretic tool for representing the Accumulating Evidence and Research Organization (AERO) within a translational trajectory. We begin in the next section by describing the basic methodology – the components of the AERO model and how these are used to construct an AERO graph. We then apply this tool to a case study involving the antibacterial agent, moxifloxacin, for the treatment of drug-susceptible tuberculosis. We close by discussing the potential utility of our approach for improving the organization, coordination, and efficiency of the drug development enterprise.

## Methods

The conceptual foundations of the AERO model emerged out of work in the philosophy of science to develop a general methodology for representing robust scientific evidence [11]. Although there are many different dimensions of robustness that figure in drug development, the version of the AERO model we present here focuses on elucidating just two of these: (1) the consistency of results among studies with an identical or similar outcome metric; and (2) the concordance of results among studies with qualitatively different outcome metrics. For example, a series of animal experiments that all show a similar effect size for a new drug is evidence of consistency. A positive direction of effect on both the surrogate outcome used in a phase 2 trial and the clinical outcome used in a phase 3 trial is evidence of concordance.

The visual structure of the AERO model is a directed acyclic graph (DAG), designed to capture these two dimensions of robustness and their relationship to three basic questions that underlie the planning of a translational research program: What is the accumulating state of total evidence? What has been the translational trajectory? What studies should be done next? We discuss each of these questions in turn, showing how they are represented in the model.

### Representing the accumulating state of total evidence

The accumulating state of evidence in a drug development trajectory is constituted by discrete experiments, proceeding from the pre-clinical *in vitro *and *in vivo* experiments to the phase 1, 2 and 3 clinical trials. In the AERO model, we represent each experiment as a node (that is, a vertex) arranged in a two-dimensional space: an x-axis, representing time, and a y-axis, representing the phase of research. The nodes are then color-coded according to the direction of their outcome: studies in support of further research (for example, positive results) are green, studies against further research (for example, negative results) are red, and studies ambivalent toward further research (for example, inconclusive results) are yellow.

**Figure 1:**
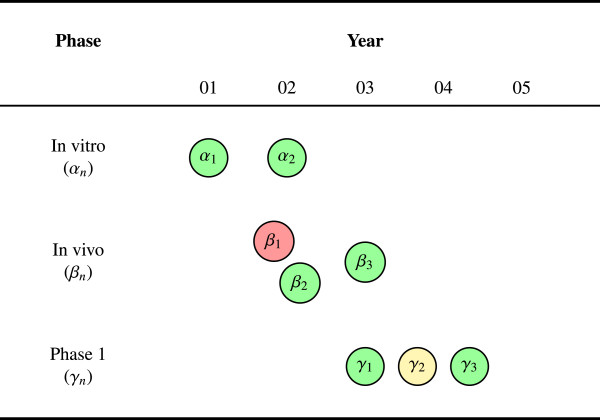
**Consistency of results. **Studies are shown as vertices. The graph shows eight experiments across five years with some degree of consistency evident at every phase of research: Both of the *in vitro *studies, two of the three *in vivo*, and two of the three phase 1 studies were positive. There is also some inconsistency within *in vivo *(*β*_1_) and phase 1 (*γ*_2_); nevertheless, the accumulating state of evidence is largely positive and the transitions between phases appear relatively smooth.

Figure 1 is an example AERO graph for a research program with eight experiments across three phases in a five-year span. Although this graph is incomplete, since it does not yet include the arrows (that is, the edges) to illustrate the translational trajectories, it nevertheless captures some features of the research program that are essential to understanding the state of total evidence. For example, we can see that the translation from animals into humans (that is, *in vivo *into phase 1) is relatively smooth. Two positive animal studies (*β*_2_,*β*_3_) suggest a potential for efficacy in humans and two positive phase 1 studies (*γ*_1_,*γ*_3_) suggest a well-tolerated drug. There is also evidence of consistency at each phase (*α*_1_ and *α*_2_, *β*_2 _and *β*_3_, *γ*_1 _and *γ*_3_). Given that consistency between experiments serves to verify findings and control for biases or random errors, which may distort the results of any single experiment, this is a desirable feature for the system to have.

Figure 1 also shows some evidence of concordance. The overall trend of the experimental findings is largely positive across each phase. With the exception of one negative *in vivo *study and one inconclusive phase 1 study, the results have all been favorable to further research. This degree of concordance is another desirable feature of the system. Depending upon the predictive power of the animal model, this pattern of robust results provides good reason for thinking that the experimental agent may be efficacious and safe.

Finally, assuming that *β*_2 _is later than *α*_2 _and *γ*_1 _is later than *β*_3_, we can see that there was a *de facto *threshold of two positive studies at each phase before proceeding to the next. These thresholds between phases are an important property of the translational trajectory, representing critical (and often expensive) decision points: When is the pre-clinical evidence sufficient to initiate human trials? When is the phase 2 evidence sufficient to justify a pivotal phase 3 trial? Many translational failures (like those mentioned above) can be understood as the result of poor or inappropriate thresholds, wherein the evidence was not sufficiently mature to warrant advancing a candidate to a later phase of research.

One plausible way to set phase thresholds is to require a certain number of positive studies at each phase before proceeding to the next. Indeed, the US FDA’s licensure requirement for two positive phase 3 trials can be thought of as just such a threshold. However, two positives is not the only viable threshold. For example, a certain number of negative studies within a phase could require either that an agent be sent back to an earlier phase or abandoned entirely. It may also be reasonable to require that there is a sufficient number of both positive and negative studies at each phase – the positive studies supporting the efficacy of the therapeutic ensemble for clinical translation and the negative studies supporting a theoretical evidence base that directly informs clinicians about how the experimental agent should not be used [12]. Leaving aside the question of which phase threshold to use, either in general or for some specific research domain, it is sufficient for the purposes here to observe that the AERO graph has the virtue of rendering each of these thresholds visually explicit.

### Representing the translational trajectory

**Figure 2:**
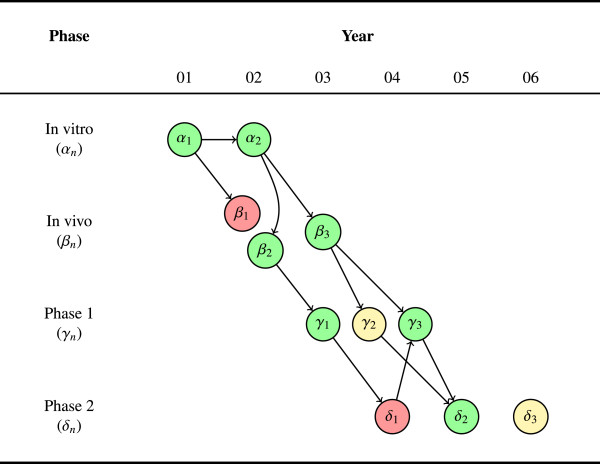
**Concordance in a trajectory. **Edges show the intellectual lineage. The graph shows eleven experiments across six years. The edges between studies represent the intellectual lineage between them and illustrate the translational research trajectories. Some trajectories are perfectly concordant (for example, *α*_1_→*α*_2_→*β*_3_→*γ*_3_→*δ*_2_), while others show discordance (for example, *α*_1_→*β*_1 _or *α*_1_→*α*_2_→*β*_2_→*γ*_1_→*δ*_1_). The phase 2 studies are also highly inconsistent (that is, one positive, one negative, and one inconclusive study), indicating a relatively rough transition into this phase. Given that phase 2 was initiated after only one positive phase 1 trial, this may indicate that the threshold of evidence used to transition into phase 2 is too low and ought to require at least some degree of consistency.

In Figure 2, we expand on Figure 1, introducing three phase 2 studies as well as arrows between studies. The arrows represent more precisely the sequence of studies and capture the intellectual lineage across the translational trajectory. For example, a phase 2 study that uses the same dosage identified in a phase 1 study should be connected by an arrow leading out from the phase 1 node and into the phase 2 node (for example, *γ*_1 _to *δ*_1_). Similarly, a phase 1 human pharmacokinetic and pharmacodynamic study should be connected to the prior *in vivo *study that identified the effective blood concentration (for example, *β*_3_ to *γ*_2 _and *γ*_3_). Borrowing terminology from the language of graph theory, we refer to studies downstream in the intellectual lineage as ‘children,’ and studies upstream as ‘parents’ (we will have more to say about how parentage is established, when we discuss the example in the next section).

This additional structure enriches judgments about consistency and concordance. Where before we could only suggest concordance by the number or ratio of positive results at each phase, now we can track a discrete translational trajectory. For example, the trajectory from *α*_1 _to *α*_2 _to *β*_2 _to *γ*_1 _is a sequence of four positive studies across three phases. Each of these studies built on the evidence in the prior study, and each showed a concordant result.

Notice, however, that the story of this trajectory gets more complicated when we follow it into phase 2. The first phase 2 study, *δ*_1_, was negative, despite following up the evidence from *γ*_1_. But negative results are not necessarily uninformative results. Thus, as the figure shows, a later positive phase 1, *γ*_3_, built upon *δ*_1_, and eventually led (in conjunction with *β*_3_), to the positive phase 2 result in *δ*_2_. The complete story of this trajectory, while not one of *perfect *concordance or consistency, can nevertheless be described as favorably robust overall.

Figure 2 also reveals that there was only positive phase 1 study (*γ*_1_) before phase 2 research began (that is, *γ*_3 _is subsequent to *δ*_1_). Again, this represents a *de facto *(if not explicit) judgment about the minimum threshold of evidence necessary to justify proceeding to the next phase. Since consistency requires at least two experiments, advancing into the next phase of research on the basis of only one experiment means that this minimum threshold included no evidence of consistency at the immediately preceding phase. For exactly the same reasons that consistency is desirable, failing to have consistency before proceeding to a subsequent phase is, in general, an undesirable feature of a research system: No verification of findings, no control for bias or random error. While a single, well-designed study may be sufficient, under particular conditions, to advance an intervention, the recent failed attempts by Bayer [13] and Amgen [14] to reproduce earlier findings demonstrates the dangers with such an approach.

Finally, we should point out the orphaned study, *δ*_3_. This node has no arrows leading into it, reflecting an experiment that is not directly justified on the basis of prior evidence within the research program. Although the general rule is probably to avoid conducting such studies, there may at times be reasons to draw on evidence or study designs that are external to the current research program. For example, the experiment may be largely based on analogical reasoning, drawing on evidence with a different drug or a different indication (the phase 3 trials of sunitinib and bevacizumab alluded to above could be described in just this way). The AERO graph makes this design choice explicit – the prudence of which will have to be judged by the fruitfulness of the subsequent research.

### Planning the next step

**Figure 3:**
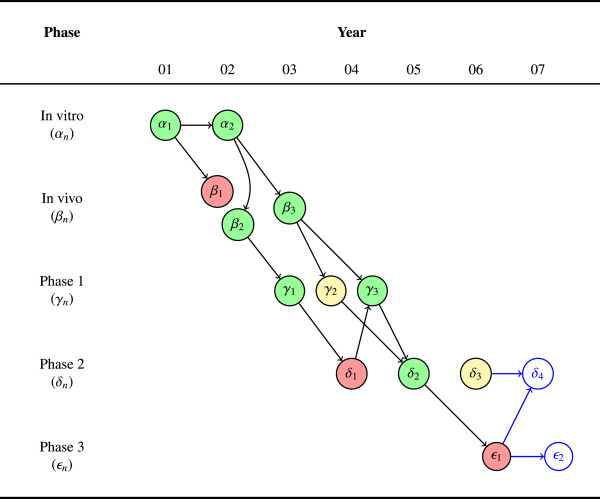
**Planning future studies. **The graph shows twelve completed experiments across six years along with two contemplated future studies, a fourth phase 2 trial (*δ*_4_) and a second phase 3 trial (*ϵ*_2_). A phase 3 trial (*ϵ*_1_) was initiated following the sole positive phase 2 study (*δ*_2_). The result of this phase 3 trial was negative, discordant with the earlier phase 2 result. Now researchers must decide which study (or studies) to do next: Trust that the accumulated evidence is still sufficient to motivate another phase 3 or return to phase 2 in search of greater consistency and a potential explanation for the discordance between *δ*_2 _and *ϵ*_1_.

The final, and perhaps most important, question we want to address with the AERO graph is: ‘What study should be done next?’ Given the state of accumulating evidence and the current trajectory, what should be the next investigation(s)? In Figure 3, we have added a single, negative phase 3 study, *ϵ*_1_, following the trajectory from *δ*_2_. We have also added two blue-lined nodes to represent the contemplated next steps.

Herein lies the real power of the AERO graph: In debating what study should come next, the visualization can sharpen the judgments of researchers, who can now pinpoint the precise translational trajectory or subset of the trajectory that they think ought to inform future research. For example, let us suppose that *ϵ*_1 _was negative for lack of clinically significant effectiveness. A researcher could argue that despite the negative result, there is still a largely positive trend (seven out of twelve studies) across the entire trajectory, the drug is very well tolerated (no negatives at phase 1), and the inconsistency across phase 2 studies has been instructive in suggesting a novel variation on the dosage and schedule that ought to be evaluated in a subsequent phase 3 trial. Yet, a different researcher may look at the same graph, identify a mechanism of action common to the inconclusive and negative studies that could explain these results, and recommend *δ*_4 _to test this hypothesis in a less costly, phase 2 trial.

The purpose of the AERO graph is thus not to replace this kind of critical thinking or eliminate disagreement about the state of total evidence. Indeed, researchers may even disagree about whether a particular study ought to be represented as positive or negative. Rather, the purpose of the AERO graph is to sharpen this disagreement by clarifying the state of evidence and translational trajectory. In other words, the prudent course of action within a clinical research program cannot be derived from the state of evidence. The decision of what to do next requires a negotiation between the practical, pragmatic, ethical, and epistemic issues at play. The representational features of the AERO model help to make the epistemic aspects of this judgment explicit.

## Results

In its *Global Plan to Stop TB 2011–2015*, the Stop TB Partnership emphasizes the need for improving coordination across the entire development and testing trajectory [10]. Moxifloxacin, an antibacterial agent in the fluoroquinolone family, is one of the new candidate drugs in their pipeline. However, as of 2010, the tuberculosis research community was confronted with a series of inconsistent results across five phase 2 studies with moxifloxacin, and disagreed about whether the state of total evidence supported moving on to conduct phase 3 trials. The results we present in this section are based on our presentation at the Tuberculosis Trials Consortium’s semi-annual meeting in October 2011, where we presented an AERO graph of moxifloxacin’s translational trajectory to help broker this dispute.

### Representing the evidence

#### Inclusion

Table 1 summarizes the results of 19 studies evaluating moxifloxacin for the treatment of drug-susceptible tuberculosis between 1998 and 2009. These studies were included based on a hand search through the citations of the published phase 2 trial reports. This list was then cross-referenced with a PubMed search using the terms ‘moxifloxacin’ and ‘tuberculosis;’ filtered by clinical trials. While this method is less rigorous than a complete systematic review, it is sufficient for the illustrative purposes of this paper. Further development on harmonizing the AERO model with the methods of systematic review is underway.

**Table 1:** Summary of moxifloxacin-TB studies

***In vitro***	**Year**	**Key**	**Outcome**	**Children**
Ji et al. [15]	1998	*u*_1_	Positive	*v*_1_
Gillespie et al. [16]	1999	*u*_2_	Positive	*w*_1_,*w*_2_
Shandil et al. [17]	2007	*u*_3_	Positive	*w*_5_,*w*_6_
***In vivo***				
Ji et al. [15]	1998	*v*_1_	Positive	*v*_2_
Miyazaki et al. [18]	1999	*v*_2_	Positive	*v*_3_,*w*_1_
Lounis et al. [19]	2001	*v*_3_	Positive	*v*_4_
Yoshimatsu et al. [20]	2002	*v*_4_	Inconclusive	*v*_5_
Nuermberger et al. [21]	2004	*v*_5_	Inconclusive	*u*_3_,*w*_3_,*w*_4_
**Phase 1**				
Gosling et al. [22]	2003	*w*_1_	Positive	*v*_5_
Pletz et al. [23]	2004	*w*_2_	Positive	*v*_5_
Gillespie et al. [24]	2005	*w*_3_	Negative	*x*_1_
Johnson et al. [25]	2006	*w*_4_	Positive	*x*_1_,*x*_2_
Nijland et al. [26]	2007	*w*_5_	Negative	–
Peloquin et al. [27]	2008	*w*_6_	Inconclusive	*x*_4_
**Phase 2**				
Burman et al. [28]	2006	*x*_1_	Negative	*w*_5_,*w*_6_,*x*_3_,*x*_4_
Rustomjee et al. [29]	2007	*x*_2_	Positive	*x*_3_
Conde et al. [30]	2009	*x*_3_	Positive	*x*_5_
Dorman et al. [31]	2009	*x*_4_	Negative	–
Wang et al. [32]	2009	*x*_5_	Positive	–

#### Extraction

**Figure 4:**
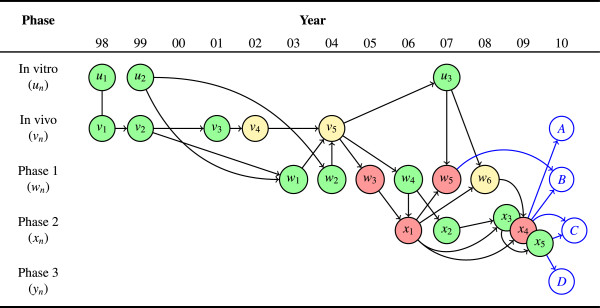
**Complete AERO graph for moxifloxacin in an anti-tuberculosis regimen.** The graph shows 19 completed experiments across 12 years along with four contemplated future studies. The overall trend of study results was positive until the transition into phase 2 (*x*_1_), when significant discordance (for example, *w*_4_→*x*_1_ and *w*_6_→*x*_4_) and inconsistency (that is, negative results in *x*_1_,*x*_4 _vs. positive results in *x*_2_,*x*_3_,*x*_5_) began to emerge. Researchers must now decide how to proceed in the face of an equivocal state of total evidence: Investigate mechanisms of discordance between animal models and human trials (*A*); investigate drug interactions (*B*); further investigate efficacy and evaluate predictivity of specific phase 2 trial designs (*C*); or proceed to a decisive phase 3 effectiveness trial (*D*).

The ‘year’ in Table 1 refers to the year of publication. The ‘key,’ generated sequentially by phase, refers to the corresponding node in Figure 4. The outcome was extracted from the published manuscript, based on the direction of the observed effect, the authors’ recommendation for further research, and any expressed qualifications or reservations. The studies listed as ‘children’ are based on a transitive reduction of the total network of citations, reconstructed to represent the historical translation of evidence as accurately as possible.

#### Non-positive outcomes

We acknowledge that the inconclusive status of the two *in vivo *studies is debatable. Despite evidence of efficacy, we nevertheless classified Yoshimatsu et al. as inconclusive due to the authors’ concerns about toxicity at the recommended dosage [20]. Nuermberger et al. did not show an improvement on their primary outcome (time-to-culture-negative), but did show a dramatic increase in early potency when moxifloxacin was substituted for isoniazid, one of the drugs in the standard regimen [21]. Gillespie et al. showed no difference in the early bactericidal activity between moxifloxacin and isoniazid [24]. Nijland et al. showed that moxifloxacin plasma concentrations are reduced when it is administered with isoniazid and rifampicin [26]. Peloquin et al. showed favorable population pharmacokinetics with moxifloxacin, but levofloxacin had the most favorable profile in their study [27]. Both Burman et al. and Dorman et al. showed no improvement in the time-to-culture-negative when moxifloxacin was substituted into the standard regimen for ethambutol and isoniazid, respectively [28,31].

#### Visual representation

Figure 4 is the complete AERO graph based on the information in Table 1 and the possible future studies, *A*…*D*. It was rendered using the *tikz *vector illustration package for LaTeX. All of the non-straight edges were shaped manually in order to aid visual comprehension. The edge between *u*_1 _and *v*_1 _is not an arrow because these two studies are published in the same report.

### Analysis

The first thing to notice about Figure 4 is how much messier is the picture of an actual translational research program compared to the toy example we discussed above. One immediate advantage of the AERO graph is that it illuminates the point about translational research: It is not a linear process, but a complex network of overlapping investigation types. Phases can even be repeated, as when evidence from a downstream phase is used to inform a subsequent upstream investigation (for example, *v*_5_ → *u*_3_).

But however descriptively accurate is this complexity, there is a prescriptive question about how organized and systematic a research program in translational medicine ought to be. Is this overlapping web of studies an intrinsic part of translational medicine? Or can it be made more orderly, with one study and one phase proceeding after another without the need to backtrack? While we will not take up this intriguing question here, it is worth pointing out that the AERO model facilitates such an inquiry.

So what can we now say about moxifloxacin’s translational trajectory? Across the entire trajectory, the trend is largely positive, with a 3:1 positive to negative ratio. There is also evidence of consistency at every phase and no negative results in the pre-clinical studies. Although to our knowledge there was no explicit evidence threshold established for this trajectory, we can nevertheless observe that for both the pre-clinical to clinical and phase 1 to phase 2 transition, the *de facto *threshold is three positive studies. Thus far, these would all seem to be encouraging properties. In fact, prior to the first negative phase 2 study in 2006, the evidence for moxifloxacin was overwhelmingly favorable.

After 2006, significant inconsistency and discordance emerges. Two of the five phase 2 studies, *x*_1 _and *x*_4_, are negative and show discordance with the earlier phase 1 and pre-clinical outcomes. Indeed, there is a subset of the total trajectory, proceeding through *w*_3 _to *x*_1 _and then *w*_5 _and *x*_4_, that is both consistently and concordantly negative. This underscores the fact that robustness is not only a property of positive results. Results can also be robustly negative.

Yet, one negative sub-trajectory is not necessarily fatal for the whole translational trajectory. In this case, we can see an overall trend toward positive outcomes, but it is not obvious that this trend is sufficient to justify a phase 3 trial. Therefore, we now consider how the AERO graph can sharpen arguments for or against each of the four proposed future studies.

#### A

The argument in favor of *A*, another *in vivo *study, is supported by the striking discordance between the pre-clinical and clinical results along the following sub-trajectory:

$$\begin{array}{rrrr} \ldots v_{5}\to w_{3}\to x_{1}\to x_{4}\Rightarrow A & & & \end{array}$$

Given that the *in vivo *models and phase 2 trials are both evaluating an efficacy endpoint, rather than the safety, pharmacokinetic, or early activity endpoints evaluated in phase 1, the discordance between *in vivo *and phase 2, combined with phase 2 inconsistency, casts a reasonable doubt on the predictive power of the animal models. For example, Dorman et al.’s (*x*_4_) hypothesis, comparing the replacement of moxifloxacin for isoniazid in the standard regimen, was informed by Nuermberger et al.’s (*v*_5_) finding in a murine model. The failure to translate this result supports revisiting the *in vivo *design and re-evaluating the import of *in vivo *experiments.

But we should note that pursuing another *in vivo *study does not necessarily invalidate the prior phase 1 or phase 2 results. Those studies can still be internally valid and their evidence can remain reliable and informative in future analyses. The rationale in favor of another animal study is that it may be more productive or efficient in the long term to try and explain the discordance between some of the extant animal and human results, rather than simply continuing to test moxifloxacin-containing regimens in humans.

#### B

The argument in favor of *B*, another phase 1 study, is supported by the inconsistencies at phases 1 and 2. In addition to the above sub-trajectory, which tracks the negative results at phase 2, this line of reasoning adds a second trajectory of emphasis:

$$\begin{array}{rrrr} \ldots v_{5}\to w_{3}\to x_{1}\to x_{4}\Rightarrow B & & & \\ \ldots x_{1}\to w_{5}\Rightarrow B & & & \end{array}$$

These sub-trajectories suggest a need to better understand the pharmacokinetic properties of moxifloxacin when used with the other drugs in the standard anti-tuberculosis regimen. Nijland et al.’s (*w*_5_) finding that some combinations with moxifloxacin produce lower plasma concentrations relate directly to this point.

#### C

The argument in favor of *C*, another phase 2 trial, emphasizes differences in experimental design across the phase 2 studies, supported by the following sub-trajectories:

$$\begin{array}{rrrr} \ldots v_{5}\to w_{4}\to x_{1}\to x_{3}\to x_{5}\Rightarrow C & & & \\ \ldots x_{1}\to x_{4}\Rightarrow C & & & \\ \ldots x_{2}\to x_{3}\to x_{5}\Rightarrow C & & & \end{array}$$

Just as we discussed a robustly negative sub-trajectory, a researcher could emphasize the robustly positive sub-trajectory and claim that if any doubts about moxifloxacin’s potential efficacy remain, that these ought to be eliminated by another, rigorous phase 2.

Importantly, the justification for *C *turns, in no small part, on assumptions about the purpose of the phase 2 trial and the threshold of evidence for proceeding to phase 3. If phase 2 trials are supposed to limit the number of candidate interventions for phase 3 strictly, then it may be reasonable to require at least a 2:1 positive-to-negative trial ratio, for example, before advancing. In which case, *C *seems a reasonable option. If, however, phase 2 trials are only supposed to rule out dangerous candidates, then three positive trials is arguably sufficient, and therefore, *C *would seem to be unnecessary.

#### D

This leads to the argument for *D*, a phase 3 trial, which could be justified on the grounds that moxifloxacin has been evaluated in three positive phase 2 trials, has not been ruled out as a novel candidate for inclusion in an effective anti-tuberculosis regimen, and is supported by the sub-trajectory:

$$\begin{array}{rrrr} \ldots v_{5}\to w_{4}\to x_{2}\to x_{3}\to x_{5}\Rightarrow D & & & \end{array}$$

As we have already mentioned, the overall picture of moxifloxacin’s trajectory is largely positive – not perfect, but arguably robust. Therefore, it may be reasonable to proceed to phase 3, despite the inconsistency across phase 2. Indeed, we should acknowledge here the REMox-TB trial (NCT00864383), an in-progress phase 3 trial evaluating a moxifloxacin-containing regimen for the treatment of drug-susceptible tuberculosis. The results of this trial, although not yet available, can be understood, in part, as an empirical test of this line of reasoning. A positive and reproducible finding in the REMox-TB trial would be evidence that the AERO graph of Figure 4 reflects a promising trajectory. Inversely, a negative finding should cast doubt on the idea that such a trajectory is robust enough to justify a phase 3 trial.

### Summary

Whichever direction is ultimately selected for future research, the AERO model can be dynamically updated in light of those new study results as they become available. For example, suppose that moxifloxacin researchers were to pursue both options *A* and *C* – another animal study and another phase 2 trial, respectively. And assume further that both of these turn out to be positive. This could potentially show that the researchers now better understand the predictive relationship between the animal and human models and perhaps finally tip the balance of phase 2 evidence clearly in favor of a moxifloxacin regimen.

Yet, even before these studies are executed, researchers could use the AERO graph to strategize about future states of evidence. In contemplating option *B*, another phase 1 trial, one might reasonably question if that result, whatever it turns out to be, would be informative and useful enough to justify the opportunity cost of not pursuing *A*, *C* or *D*.

We should also acknowledge that these alternative research strategies are neither exhaustive nor mutually exclusive. A well-funded research program may be able to pursue all of these and other options simultaneously. The aim in this section is simply to show how the AERO graph can help to clarify and sharpen the rationale for the various possibilities and in so doing, aid decision-making about the directions for further research.

That said, the more fundamental questions elucidated by the moxifloxacin trajectory – calling into question the predictive value of the animal model, reconsidering the purpose for phase 2, and the appropriate thresholds of robustness at each phase transition – should not be overlooked, even if the decision is made to proceed to a phase 3 trial. Part of what Figure 4 illustrates is that there are many unanswered questions about moxifloxacin’s trajectory and the underlying causal relationships, and until at least some of these are addressed, a phase 3 trial, whatever its outcome, will be less informative that it could be, and hence, reflect an inefficient research strategy over the long term.

## Discussion

Stepping back from this specific case study, there are a number of possible elaborations and applications of the AERO modeling approach that are worth discussing. To begin with, the color-coding we presented here, which classifies studies as positive, negative, or inconclusive, has significant virtues of simplicity and ease of interpretation, but this is far from the only option. For example, a continuous shading scale could be used to represent the effect size or precision of each study; or a five-point ordinal scale could be used, corresponding to the region of posterior interval. The fundamental structure of the AERO model is the directed acyclic graph with study-type strata; the other graphical properties can be extensible in any way that supports decision-making.

The AERO graph can also be thought of as reflecting the maturity of causal knowledge within a given research domain. A robust field of mostly green nodes would suggest that investigators have full command of the mechanisms in the causal system, whereas a field of predominantly red or yellow nodes would suggest utter lack of contact with the causal factors. A pattern of pre-clinical green that consistently turns to red in clinical translation should cast doubt on the validity of pre-clinical models. A thin thread of green would perhaps suggest just a lucky find, while an evenly balanced network of green and red nodes would suggest a new, emerging domain with which investigators may have only a limited understanding.

This relates to another extension of the approach – comparing and contrasting multiple trajectories. We could see such a comparative analysis being useful in a number of ways: For example, the AERO graph of a successfully translated agent could be used as the model for how new agents in a treatment domain ought to be vetted; or a population of AERO graphs could elucidate systematic differences in efficiency or risk and benefit across different research domains.

Looking ahead to envision the potential utility of the AERO model, particularly as a means to improve the efficiency and coordination of the drug development enterprise, we believe that publications and protocols could both benefit from including an AERO graph. Just like other kinds of visual tools, such as Thorpe et al.’s PRECIS graph [33] or Langan et al.’s graphical augmentation to the meta-analytic funnel plot [34], serve to aid judgments about the quality and direction of evidence, so too can the AERO model help to make invisible or inchoate properties of the research system explicit.

Indeed, AERO graphs could provide a convenient check for investigators, institutional review boards, journal editors, and physicians. The comprehensibility of the representation allows for anyone even modestly familiar with a particular domain to be able to compare and contrast alternative representations. As a consequence, a report that ignores a substantial portion of the available evidence can be more easily detected, since its representation will differ dramatically. This is not to contradict what we claimed earlier about disagreement, as different researchers may have equally legitimate interpretations of their field. However, the apparent contrast between interpretations of the evidence will demand an explanation for why the authors represented the field in one way rather than another.

## Conclusion

The AERO model provides an immediate visual representation of the number of studies done at any mode, depicting both the direction of evidence and the relationship of each study to the larger translational trajectory. In so doing, it helps to address the widespread concerns about efficiency, coordination, and organization in translational medicine. To be sure, the visual representation does not capture all of the available information about a research program. For example, the details of differing experimental designs or conduct may be relevant to understanding failures of robustness. Just as we showed in the previous section, the argument for one course of action over another relies upon knowledge of these additional details about each experiment. Nevertheless, the AERO model provides a systematic representation that is capable of sharpening these judgments and revealing some of the existing patterns across a translational trajectory.

We recognize that the approach we have presented here is but a preliminary sketch. While we have applied the AERO model to a single case study, much more work is needed to develop the approach to its full potential. But particularly given the stakes, thinking about ways to better analyze and judge the structure of clinical research programs as a whole seems a vital line of inquiry. The AERO model is one piece of this inquiry.

## Abbreviations

AERO: Accumulating Evidence and Research Organization; DAG: Directedacyclic graph.

## Competing interest

The authors declare that they have no competing interests.

## Authors’ contributions

All authors contributed to the conception and design of the manuscript. SPH wrote all drafts. CMH and CW commented and suggested changes on all drafts. All authors read and approved the final manuscript.
